# Dietary Marine Oils Selectively Decrease Obesogenic Diet-Derived Carbonylation in Proteins Involved in ATP Homeostasis and Glutamate Metabolism in the Rat Cerebellum

**DOI:** 10.3390/antiox13010103

**Published:** 2024-01-15

**Authors:** Francisco Moreno, Lucía Méndez, Ana Raner, Bernat Miralles-Pérez, Marta Romeu, Sara Ramos-Romero, Josep Lluís Torres, Isabel Medina

**Affiliations:** 1 Instituto de Investigaciones Marinas—Consejo Superior de Investigaciones Científicas (IIM-CSIC), Eduardo Cabello 6, E-36208 Vigo, Spain; fmoreno@iim.csic.es (F.M.); ana0raner@gmail.com (A.R.); medina@iim.csic.es (I.M.); 2 Universidad de Vigo, Circunvalación ao Campus Universitario, E-36310 Vigo, Spain; 3 Unidad de Farmacología, Facultad de Medicina y Ciencias de la Salud, Universidad Rovira i Virgili, Sant Llorenç 21, E-43201 Reus, Spain; bernat.miralles@urv.cat (B.M.-P.); marta.romeu@urv.cat (M.R.); 4 Department of Cell Biology, Physiology and Immunology, Faculty of Biology, University of Barcelona, Av Diagonal 643, E-08028 Barcelona, Spain; sara.ramosromero@ub.edu; 5 Nutrition & Food Safety Research Institute (INSA-UB), Maria de Maeztu Unit of Excellence, E-08921 Santa Coloma de Gramenet, Spain; joseplluis.torres@iqac.csic.es; 6 Instituto de Química Avanzada de Catalunya—Consejo Superior de Investigaciones Científicas (IQAC-CSIC), Jordi Girona 18-26, E-08034 Barcelona, Spain

**Keywords:** omega-3 fish oil, high-fat and high-sucrose diet, prediabetes, protein carbonylation, cerebellum, oxidative stress, marine natural antioxidants

## Abstract

The regular intake of diets high in saturated fat and sugars increases oxidative stress and has been linked to cognitive decline and premature brain aging. The cerebellum is highly vulnerable to oxidative stress and thus, obesogenic diets might be particularly detrimental to this tissue. However, the precise molecular mechanisms behind obesity-related brain damage are still not clear. Since protein carbonylation, a biomarker of oxidative stress, influences protein functions and is involved in metabolic control, the current investigation addressed the effect of long-term high-fat and high-sucrose diet intake on the cerebellum of Sprague-Dawley rats by deciphering the changes caused in the carbonylated proteome. The antioxidant effects of fish oil supplementation on cerebellar carbonylated proteins were also investigated. Lipid peroxidation products and carbonylated proteins were identified and quantified using immunoassays and 2D-LC-MS/MS in the cerebellum. After 21 weeks of nutritional intervention, the obesogenic diet selectively increased carbonylation of the proteins that participate in ATP homeostasis and glutamate metabolism in the cerebellum. Moreover, the data demonstrated that fish oil supplementation restrained carbonylation of the main protein targets oxidatively damaged by the obesogenic diet, and additionally protected against carbonylation of several other proteins involved in amino acid biosynthesis and neurotransmission. Therefore, dietary interventions with fish oils could help the cerebellum to be more resilient to oxidative damage. The results could shed some light on the effect of high-fat and high-sucrose diets on redox homeostasis in the cerebellum and boost the development of antioxidant-based nutritional interventions to improve cerebellum health.

## 1. Introduction

The regular intake of diets high in saturated fat and sugars is identified as the primary cause of the widespread prevalence of obesity and overweight, which have reached pandemic levels in the 21st century [1]. These conditions are major risk factors for various chronic diseases, including diabetes, cardiovascular diseases and cancer [2]. Additionally, obesogenic diets may contribute to cognitive impairment during aging and accelerate the progression of neurodegenerative diseases [3]. It has been found that the long-term intake of high-fat diets inhibits purinergic and cholinergic functions in the central nervous system, which may accelerate the progressive memory loss, language decline and other cognitive disruptions observed in Alzheimer’s disease (AD) [4]. Moreover, the consumption of these obesogenic diets has unequivocally been associated with heightened oxidative stress [5]. From a mechanistic perspective, the connection between obesogenic diets and these health issues lies in oxidative stress—an imbalance between oxidants and antioxidants that favors oxidants. This disequilibrium leads to a disruption of redox signaling and control, and/or molecular damage [6].

The central nervous system (CNS) is highly sensitive to oxidative stress due to its substantial oxygen consumption (constituting 20% of the total basal oxygen budget) used to support ATP-intensive neuronal activity. This sensitivity is further compounded by the enrichment of unsaturated fatty acids, a high concentration of redox-active transition metals and mitochondria, and a modest antioxidant defense, among other reasons [7]. Within the CNS, certain regions are more susceptible to oxidation than others. Notably, the cerebellum stands out as one of the specific areas particularly prone to oxidative stress [8]. Consequently, it becomes especially vulnerable to the oxidative insult stemming from the consumption of obesogenic diets. The cerebellum plays a pivotal role in various essential functions for human health. Apart from its traditional motor functions, which involve balance maintenance and movement coordination, the cerebellum is integral to processes such as cognition, learning, memory and emotional regulation [9].

Oxidative stress induces damage to nucleic acids (DNA and RNA), lipids and proteins. Specifically, protein carbonylation, the main hallmark of protein oxidation, serves as a reliable biomarker for oxidative stress in various neuropathologies. This reliability arises from the general stability of oxidized proteins as compared to other oxidation products [10]. Protein carbonylation is a non-enzymatic and irreversible post-translational modification (PTM) that involves the introduction of a reactive carbonyl moiety, such as an aldehyde, ketone or lactam, into a protein [11]. Protein-bound carbonyls can result from: (a) the direct attack of reactive oxygen species (ROS) on the side chains of several amino acids (proline, arginine, lysine, threonine and tryptophan); (b) oxidative cleavage of proteins via the α-amidation pathway or oxidation of glutamine side chains; (c) the secondary reaction of cysteine, histidine, lysine and arginine with reactive carbonyl species (RCS) derived from lipid peroxidation (advanced lipoxidation end products (ALEs)) or produced by the reaction to reducing sugars or their oxidation products (advanced glycation end products (AGEs)) [12]. Protein carbonylation usually triggers functional losses, fragmentation and enhanced susceptibility to proteolytic digestion. Carbonylation eventually leads to protein aggregation and proteotoxic stress, which, in turn, increases oxidative stress and causes mitochondrial dysfunction. Therefore, protein carbonylation induces a loss of proteostasis (protein homeostasis), one of the main hallmarks of the aging process [13]. These events ultimately provoke neuronal death, the leading cause of neurodegenerative diseases [10,14]. Additionally, protein carbonylation and inflammation are closely interconnected. Carbonylated proteins can function as damage-associated molecular patterns (DAMPs), interacting with pattern recognition receptors (PRRs) and activating the innate immune system [15].

Protein carbonylation is, in fact, very selective. The vulnerability of each protein to carbonylation in vivo depends on multiple factors, including amino acid sequence and three-dimensional conformation, protein function, concentration, cellular location and the specific oxidative environment. This selectivity makes carbonylation crucial in metabolic control systems [16], especially in the CNS, where multiple processes are mediated by redox signaling [7]. Previous studies revealed that obesogenic diets selectively increased carbonylation of proteins participating in metabolic pathways crucial to liver and kidney function [17,18]. Recent findings have demonstrated that the intake of a high-fat and high-sucrose diet results in increased carbonylation of specific target proteins in the brain cortex of rats [19]. While some studies have reported elevated levels of oxidative protein damage in the cerebellum due to high-fat diet consumption [20,21], the carbonylated proteome of the cerebellum remains largely unexplored. More comprehensive knowledge about the precise molecular and cellular mechanisms affected by the intake of obesogenic diets is essential for a thorough understanding of their deleterious effects.

In this context, nutritional strategies centered on antioxidants used to promote brain health have garnered significant attention in recent decades. The list of antioxidant compounds used to prevent age-related cognitive impairment is large, encompassing vitamins C and E, zinc, carotenoids, polyphenols and more [22]. Some of them, such as olive polyphenols [23], thymoquinone [24], melatonin [25], agavins [26] and gastrodin [27] have been explored for enhancing cerebellum performance.

Among nutrients with antioxidant properties, the ω-3 polyunsaturated fatty acids (ω-3 PUFAs) are notable due to their distinctive attributes, in particular eicosapentaenoic acid (EPA) and docosahexaenoic acid (DHA), which are naturally present in fish and other seafood products. ω-3 PUFAs are good candidates for promoting brain health as they are crucial for the correct functioning of the CNS. The brain is the major reservoir for ω-3 PUFAs, with their concentration depending on dietary intake and their adequate transport through the blood–brain barrier (BBB) [28]. Any deficiency in ω-3 PUFAs, especially DHA, can lead to brain malfunctioning. This is because they are involved in multiple brain processes, such as neurotransmission, synaptic plasticity, neurogenesis and neurodegeneration [29].

Furthermore, ω-3 PUFAs also play a critical role in neuroinflammation and oxidative stress. On one hand, they are precursors of a variety of lipid mediators with potent anti-inflammatory and pro-resolving effects, likely playing several other roles in multiple signaling pathways [30]. On the other hand, ω-3 PUFAs exhibit antioxidant properties by regulating the nuclear factor erythroid 2 like 2 (NFE2L2) and heme-oxygenase-1 (HO-1), activating antioxidant enzymes and significantly increasing the production of reduced glutathione (GSH) [22].

The capacity of ω-3 PUFAs to protect the brain from oxidative stress has been described in several animal models [31,32,33,34]. Fish oil supplementation also demonstrated antioxidant properties and selective protection against carbonylation of specific proteins in the livers [17] and kidneys [18] of rats that had been fed an obesogenic diet. Very recently, similar effects were observed in the brain cortex [19]. Whether fish oil supplementation could counteract the effect of obesogenic diets by selectively modulating the carbonylated proteome in the cerebellum remains unknown.

Hence, the current research aims to achieve two primary objectives: (1) deciphering the effects of the long-term feeding of a high-fat and high-sucrose (HFHS) diet on the carbonylated proteome in the cerebellum of male Sprague–Dawley rats; and (2) testing whether supplementation of the diet with a fish oil containing 50% of its fatty acids as EPA and DHA can restrain the oxidative damage caused to the cerebellar proteome by the obesogenic diet. For the comprehensive evaluation of the cerebellar carbonylated proteome, the research uses a redox proteomic approach for the identification and quantification of the target proteins of carbonylation, and the evaluation of the metabolic and signaling pathways modulated by the diet and the supplementation through changes in this oxidative PTM in the rat cerebellum.

## 2. Materials and Methods

### 2.1. Description of the Dietary Intervention with Fish Oils and Characterization of the Diet-Induced Prediabetes Animal Model

The cerebellar tissue used for this study comes from the same set of animals that was used in previous analyses [17,18,19,35,36]. Briefly, the experiments were carried out on male Sprague–Dawley rats (*n* = 36; aged 8–9 weeks in the beginning of experiment). The rats were randomly divided into four dietary groups (*n* = 9): (a) control standard diet group (STD-C), which was fed a standard diet (Teklad Global 14% Protein Rodent Maintenance Diet, Harlan Laboratories, Derby, UK); (b) fish oil-supplemented STD diet group (STD-ω3), which was fed the STD diet supplemented with a fish oil containing 50% fatty acids as a mix of EPA and DHA in 1:1 ratio; (c) control high-fat high-sucrose diet group (HFHS-C), which was fed an obesogenic diet (TD.08811 45% kcal Fat Diet, Harlan Laboratories, Derby, UK); (d) fish oil-supplemented HFHS diet group (HFHS-ω3), which was fed the HFHS diet supplemented with the fish oil (HFHS-ω3). A supplement of soybean oil was added to STD-C and HFHS-C control groups to equilibrate caloric intake and the influence of experimental intervention between groups. Supplements were provided by oral gavage (0.8 mL/Kg body weight/week) for 21 weeks. The cerebellum was finally extracted at 4 °C, washed, weighed and immediately frozen with liquid nitrogen. Samples were kept at −80 °C until used.

Detailed information on diet composition and fatty acid diet profiles are provided in Tables S1 and S2, respectively. Additionally, details about the health status of the rats and other experimental conditions can be found in the previous publications [17,18,19,35,36], with a summary of the most relevant data for the current investigation presented in Table S3.

The animal experiments rigorously adhered to the European Union guidelines for the care and management of laboratory animals. The Research Council (CSIC) Subcommittee of Bioethical Issues (ref. AGL2013-49079-C2-1-R) reviewed and approved all animal procedures; the regional Catalan authorities (reference no. DAAM7921) licensed them.

### 2.2. Fatty Acid Profile Analysis

Lipid extraction for the analysis of cerebellum fatty acid profiles was performed according to the Bligh and Dyer method [37], using the extraction solvent dichlorometane: methanol: water in a 2:2:1 ratio (*v*/*v*). Total lipid content was measured using gravimetric quantification in a Sartorius Precision Balance (2023, Sartorius AG), and values were normalized by the total tissue weight (g). The Lepage and Roy method [38] was followed to obtain the fatty acid profiles in the cerebellum. The organic phase (0.6 mg) was transesterified and analyzed via gas chromatography (GC/FID, Clarus 500, Perkin–Elmer, Waltham, MA, USA). The internal standard used for quantification was nonadecanoic acid (19:0).

### 2.3. Lipid Peroxidation Product Analysis

To measure the levels of conjugated dienes hydroperoxides, the method described by the American Oil Chemists’ Society (AOCS) [39] was followed, using the organic fraction obtained after the Bligh and Dyer protocol described in Section 2.2.

To measure oxidized phospholipids (oxPL), 4-hydroxynonenal (4-HNE) and malondialdehyde (MDA), dot-blot immunoassays were conducted. Measures of these lipid peroxidation products included both the unbound fraction and the fraction bound to proteins, although the protocol was essentially the same. The unbound fraction was measured in the lipid extract produced using the Bligh and Dyer protocol. To determine the protein-bound fraction, the protein pellets resulting after the Bligh and Dyer extraction were resuspended in 7 M urea, 2 M thiourea, 2% CHAPS and 0.4% DTT, and protein concentration was calculated using the Bradford’s method [40].

For dot-blot analysis, either 5 μg of lipid extract or protein sample were blotted onto PVDF membranes. Samples were left to dry, and then the membranes were blocked for 1 h at room temperature. After blocking, membranes were incubated overnight at 4 °C with primary antibodies in a blocking buffer, namely anti-4-HNE (1:1400), anti-MDA (1:20,000) and anti-oxPL (1:500). The 4-HNE- and MDA-membranes were incubated for 1 h at room temperature with the proper secondary anti-bodies FITC-labeled (1:1000) in a blocking buffer. The HNE- and MDA-membranes were then washed and exposed to UV light in the UVP BioDoc-It2 Gel Imaging System UV transilluminator (Analytik Jena AG, Upland, CA, USA) with a 520-nm band-pass filter (520DF30 62 mm). Since the primary antibody anti-oxPL was already labeled (TopFluor^®^-labeled), the oxPL-membranes were simply washed and directly exposed to UV light.

### 2.4. Total and Individual Protein Carbonylation Analysis

Proteins were extracted from 200 mg of the cerebellum after homogenization (Ultra Turrax© T10 high-performance disperser, IKA^®^-Werke GmbH & Co. KG, Staufen, Germany) of the tissue in 25 volumes (*w*/*v*) of 20 mM sodium phosphate buffer pH 6.0, 1 mM EDTA, 0.5 mM MgCl_2_, 10 μL/mL of Proteoblock^TM^ Protease Inhibitor Cocktail and 5 mM of PMSF). The cytosolic protein fraction was recovered in the supernatant after centrifugation at 100,000× *g* (60 min at 4 °C). The myofibrillar protein fraction was obtained via homogenization of the protein pellets in 10 volumes of 10 mM Tris-HCl buffer pH 7.2, 0.6 M NaCl, 10 μL/mL of Proteoblock^TM^ Protease Inhibitor Cocktail and 5 mM of PMSF. The final homogenates were centrifuged at 16,000× *g* (15 min at 4 °C) to recover the supernatants, which contained the myofibrillar proteins. Both cytosolic and myofibrillar protein fractions were quantified by the BCA method [41] and stored at −80 °C until used.

Carbonylated proteins in the cerebellum were labeled after incubation with 1 mM of fluorescein thiosemicarbazide (FTSC) at 37 °C for 2.5 h in the dark. Then, the proteins were precipitated with 20% of trichloroacetic acid (TCA) (*w*/*v*), centrifuged (16,000× *g*, 10 min, 20 °C), and the excess of FTSC was removed by washing the protein pellets with a mixture of ethanol/ethyl acetate 1:1. The protein pellets were finally resolubilized in 7 M urea, 2 M thiourea, 2% Chaps, 0.5% Pharmalyte 3–10, 0.5% IPG 3–10 buffer and 0.4% DTT, and the Bradford’s method [40] was used for protein quantification.

For total protein carbonylation analysis, 10 μg of labeled samples was resolved in monodimensional (1-D) 10–12% SDS–polyacrylamide gel electrophoresis (PAGE) (Mini-PROTEAN 3 cell, Bio-Rad, Hercules, CA, USA).

For specific protein carbonylation analysis, 100 μg of labeled samples was resolved in bidimensional (2-D) electrophoresis gels. The proteins were resolved according to their isoelectric point in the first dimension, using 7-cm IPG dry strips with a pH range of 3 to 10 in the PROTEAN i12 IEF System (Bio-Rad) working at 20 °C. Proteins were resolved according to their mass in the second dimension by placing the IPG strips (reduced and alkylated) onto 12% SDS–PAGE gels (Mini-PROTEAN 3 cell, Bio-Rad, Hercules, CA, USA).

The labeled proteins resolved in 1D and 2D gels were exposed to UV light in the UVP BioDoc-It2 Gel Imaging System UV transilluminator (Analytik Jena AG, Upland, CA, USA) with a 520-nm band-pass filter (520DF30 62 mm) to visualize the carbonylated proteins. The gels were then stained overnight with Coomassie dye PhastGel Blue R-350 (GE Healthcare Bio-Sciences AB, Uppsala, Sweden) to reveal the total protein amount. The FTSC-labeled protein signal was normalized using the Coomassie-stained protein signal [19].

### 2.5. Identification of the Carbonylated Proteome and Functional Enrichment Analysis

For protein identification, 500 μg of FTSC-labeled protein sample was resolved in 18-cm IPG dry strips with a pH range of 3 to 10 (Ettan IPGphor II isoelectric focusing system, GE Healthcare Bio-Sciences AB, Uppsala, Sweden)) at 20 °C. The strips were reduced and alkylated and placed onto 12% SDS–PAGE gels to run the second dimension at 15 °C (Ettan DALTsix electrophoresis system, GE Healthcare Bio-Sciences AB (Uppsala, Sweden). Spots containing carbonylated proteins were manually excised while gels were exposed to UV light in the UVP BioDoc-It2 Gel Imaging System UV transilluminator (Analytik Jena AG, Upland, CA, USA), equipped with a 520-nm band-pass filter (520DF30 62 mm) and digested with 0.5 μM trypsin in 50 mM NH_4_HCO_3_ buffer, pH 8 (overnight at 37 °C).

Then, 3 μL of the tryptic-digested sample was subjected to nano-LC ESI-IT-MS/MS analysis using a Dionex UltiMate 3000 Series chromatographer (ThermoFisher, Rockford, IL, USA) coupled with a dual-pressure linear ion trap mass spectrometer LTQ Velos Pro with electrospray ionization (ESI) (Thermo Fisher Scientific, Inc., Rockford, IL, USA). The digested sample was concentrated and washed in a μ-Precolumn C18 PepMap, 300 μm i.d. × 5 mm (Thermo Scientific, San Jose, CA, USA), using mobile phase A (0.1% formic acid in water) and a 10 μL/min flow rate. Then, tryptic peptides were resolved on an analytical C18 column (Acclaim PepMap RSLC C18, 2 μm, 100 Å, 75 μm i.d. × 15 cm, Thermo Scientific, San Jose, CA, USA) working at a 300 nL/min flow rate in a 20-min linear gradient elution from 5% to 40% of mobile phase B (0.1% formic acid in acetonitrile).

Separated peptides were then subjected to tandem mass spectrometry (MS/MS) analysis working in data-dependent acquisition (DDA) and positive mode. Data acquisition and instrument control were performed with Xcalibur 2.0 and Tune 2.2 software (Thermo Fisher Scientific, Inc., Rockford, IL, USA). MS1 scans were acquired from 400 to 1600 Da, and the subsequent MS2 analysis was done with the six most intense precursor ions with ≥+2 charge state. Precursor ion fragmentation was performed in collision-induced dissociation (CID) mode, with 35% of normalized collision energy, 2 Da of isolation width and 30 s of dynamic exclusion after the second fragmentation event.

Carbonylated protein identification was finally achieved when the raw files obtained in the MS/MS analysis were searched against the Uniprot/Swiss–Prot *Rattus norvegicus* database using the PEAKS DB tool of the PEAKS Studio software version 7.0 (Bioinformatics Solutions Inc., Waterloo, ON, Canada). The following search criteria were set: trypsin as proteolytic enzyme, carbamidomethylation of cysteine and oxidation of methionine, histidine and tryptophan as variable modifications, a maximum of 2 missed cleavages/peptide and ±1 Da and ±0.6 Da of mass tolerance for precursor and product ion scans, respectively. Proteins were considered successfully identified if the false discovery rate (FDR) was lower than 0.1%.

For the functional enrichment analysis, the Uniprot code of the carbonylated proteins identified in the rat cerebellum were submitted to the STRING (Search Tool for the Retrieval of Interacting Genes) software version 11.5 (http://stringdb.org/, available freely online, accessed on 1 September 2023), choosing Rattus norvegicus as the organism. The Gene Ontology (GO) terms of cellular component, molecular function and biological processes, and the Kyoto Encyclopedia of Genes and Genomes (KEGG) pathways with an FDR < 0.05, corresponding to the *p*-value corrected for multiple testing using the Benjamini–Hochberg procedure [42], were considered significantly enriched in carbonylated proteins.

### 2.6. Densitometric Image Analysis

1D gel images were analyzed with the LabImage 1D software version 16.04.08 (Kapelan Bio-Imaging Solutions, Halle, Germany). Dot-blot membrane images and 2D gel images were analyzed with the PDQuest software version 7.4 (Bio-Rad, Hercules, CA, USA).

### 2.7. Statistical Analysis

Statistical analysis of the data was performed with Jamovi Desktop version 2.3.18.0 (available for free download from https://www.jamovi.org, accessed on 19 November 2023). Data were tested for normal distribution and homogeneity of variance using Shapiro–Wilk’s and Levene’s tests, respectively. When both assumptions were met, the effects of the diet (D) and the supplement (S) or their interaction (D × S) were analyzed using the two-way Analysis Of Variance (ANOVA) test. When parametric assumptions were violated, data were analyzed with the nonparametric Kruskal–Wallis test. Data are mean and standard deviation (SD). The pairwise comparisons of the means were conducted with the post hoc test Tukey HD. The significance level was fixed at *p* < 0.05 in all the tests.

### 2.8. Materials and Reagents

The fish oil was a mixture of AFAMPES 121 EPA (AFAMSA, Vigo, Spain) and EnerZona Omega 3 RX (Milan, Italy). The soybean oil was from Clearspring Ltd. (London, UK).

Antibodies.com (Europe AB, Stockholm, Sweden) supplied the primary antibodies anti-4-HNE (mouse monoclonal (HNEJ-2) antibody to 4-HNE) and anti-MDA (goat polyclonal antibody to malondialdehyde), and the secondary antibodies goat anti-mouse IgG (H + L) (FITC) and donkey anti-goat IgG (H + L) (FITC). The TopFluor^®^ labeled E06 mouse monoclonal antibody (IgM), which targets anti-oxidized phospholipid (oxPL), was purchased from Avanti^®^ Polar Lipids, Inc. (Croda international Plc, Birmingham, AL, USA).

The PVDF membranes, Bio-Rad protein assay and acrylamide and bis-N,N-methylene-bis-acrylamide were bought from Bio-Rad Laboratories (Hercules, CA, USA). GE Healthcare Bio-Sciences AB (Uppsala, Sweden) provided the IPG buffer 3–10 pH and pharmalyte 3–10 pH, and the IPG strips Immobiline DryStrip gels for isoelectric focusing (IEF) of pH range 3–10 and lengths of 7 and 18 cm. The bromophenol blue PlusOne was obtained from Cytiva (Marlborough, MA, USA). The fluorescein-5-thiosemicarbazide (FTSC) was bought from Invitrogen (Carlsbad, CA, USA), the trypsin sequencing grade used for protein digestion from Promega (Madison, WI, USA) and the protease inhibitor ProteoBlock from Thermo Fisher Scientific Inc. (Rockford, IL, USA).

The reagents obtained from Sigma (St. Louis, MO, USA) were bicinchoninic acid (BCA) assay, 3-((3-cholamidopropyl) dimethylammonio)-1-propanesulfonate (CHAPS) detergent, ethylenediaminetet-raacetic acid (EDTA), dithiothreitol (DTT), iodoacetamide (IA), phenylmethylsulfonyl fluoride (PMSF), Tris Hydrochloride (Tris–HCl), N,N,N′,N′-Tetramethyl ethylenediamine (TEMED) and trichloroacetic acid (TCA). Serva (SERVA Electrophoresis GmbH, Heidelberg, Germany) provided the BlueBlock Blocking Solution, Phosphate-Buffered Saline (PBS) 10× solution and the PBS with 0.05% TweenTM-20 (PBST) 10× solution.

## 3. Results and Discussion

### 3.1. Effects of the High-Fat and High-Sucrose Diet and Fish Oils on Lipid and Protein Oxidation in the Cerebellum

The long-term intake of the HFHS diet induced a prediabetic state in rats, accompanied by systemic oxidative stress (Table S3). Prediabetic rats exhibited higher levels of protein carbonylation in the plasma, liver and kidneys [17,18,36]. The consumption of the HFHS diet also led to an increased accumulation of lipid peroxidation product and carbonylated proteins in the brain cortex [19]. The current results revealed that the HFHS diet elevated the oxidative stress in the cerebellum of prediabetic rats as well. As shown in Table 1, the cerebellum of HFHS-C rats accumulated significantly more lipid peroxidation products and oxidized proteins than STD-C rats.

Regarding lipid peroxidation, the intake of the HFHS diet significantly increased formation of MDA in the rat cerebellum as compared to the STD-C group, while no changes in conjugated dienes values were detected between diets. Therefore, the effects of the HFHS diet were manifested in the end products of lipid peroxidation rather than intermediate ones.

The significant accumulation of oxidized phosphatidylcholines (oxPC) in the cerebellum of HFHS-fed rats is noteworthy, because of their defined role as biomarkers of oxidative stress and as DAMP, implicated in the modulation of neuroinflammatory processes [43].

As for protein oxidation, the HFHS diet significantly elevated the formation of HNE and MDA-protein adducts and the levels of protein carbonylation in both cytosolic and myofibrillar cerebellar protein fractions.

Hence, the data indicated that the cerebellum was highly vulnerable to oxidative stress induced by the consumption of the obesogenic diet. These findings align with existing literature, which demonstrates the cerebellum’s particular sensitivity to oxidative stress [8]. The vulnerability to oxidative stress of the cerebellar granule neurons is so pronounced that even exposure to oxygen tension in ambient air (∼20%) under in vitro cell culture conditions increases cell death [44].

Previous studies have reported that the intake of high-fat diets increases the accumulation of carbonylated proteins [26] and lipid peroxidation products [24] in both cerebellar granule neurons and Purkinje cells. In these studies, the accumulation of oxidized molecules precipitated degeneration and cell loss. The loss of cerebellar neurons has been associated with a decline in cognitive functions [45] and impaired motor coordination [46,47]. Neuronal losses in the cerebellum also occur during aging [48,49] and in numerous neurological diseases [50], all characterized by elevated levels of oxidative stress [51]. This underscores the link between oxidative stress and neuronal damage, emphasizing the imperative for further investigation into this critical topic. Additionally, it has been documented that metabolic diseases like diabetes induce a loss in the number and size of neurons in cerebellum [52]. Elevated levels of insulin may be especially deleterious for the cerebellum, a structure of the CNS particularly sensitive to insulin action, as the insulin receptor is strongly expressed in the cerebellar cortex [53]. Although the precise mechanisms of the detrimental effect of insulin action on the cerebellum are not fully understood, oxidative stress seems to be definitively involved [54]. Accordingly, the current study demonstrated that elevated levels of insulin in plasma (Table S3) concurs with the accumulation of oxidized lipids and proteins in the cerebellum.

The supplementation of the HFHS diet with a fish oil rich in EPA and DHA ameliorated the prediabetic state of the rats and significantly decreased general oxidative stress [17,18,36]. Given these findings and the recent demonstration of the capacity of the fish oil to counteract oxidative damage in the brain cortex [19], the current study aimed to investigate if fish oil could modulate the oxidative stress in the cerebellum of the rats as well.

Measures of oxidative stress biomarkers in HFHS-fed rats, supplemented with fish oil (Table 1), indicated that fish oil alleviated the diet-induced oxidative stress in the rat cerebellum. Thus, HFHS-ω3 rats showed significantly lower levels of lipid peroxidation (significantly fewer oxPC, and MDA and HNE adducted to cerebellar proteins) and protein carbonylation. As shown in Table 1, cytosolic proteins were more sensitive to the fish oil antioxidant effect and significantly decreased their carbonylation level. The myofibrillar protein fraction mirrored this trend.

The antioxidant effect of the fish oil supplementation in the cerebellum was likely mediated by the significant enrichment in ω-3 PUFAs and the concurrent decrease in ω-6 PUFAs (Table 1), creating an anti-inflammatory environment. Furthermore, the decrease in insulin levels induced by fish oil supplementation (Table S3) could also contribute to its positive effects, especially considering the cerebellum’s high sensitivity to insulin, as previously discussed. Interestingly, fat-1 transgenic mice, which endogenously synthesize ω-3 PUFAs, demonstrated protection from Purkinje cell loss in the cerebellum when subjected to streptozotocin-induced diabetes [55].

In the standard dietary context, fish oil supplementation did not significantly alter the tested biomarkers of oxidative stress (Table 1). However, some biomarkers, like oxPC and protein carbonyls, showed a tendency to increase, albeit not significantly, in the STD-ω3 group as compared to the STD-C group. In any case, the oxidative stress biomarkers in STD-ω3 rats remained significantly lower than in HFHS-C rats. This slight elevation of oxidative stress in the STD dietary context aligns with systemic redox data (Table S3) and may be attributed to the significant enrichment of cellular membranes in ω-3 PUFAs (Table 1). Hypothetically, under healthy dietary conditions, the moderately increased ROS production from ω-3 PUFAs could induce antioxidant signaling, preparing the cell to be more resilient against potential oxidative insults [22,56].

### 3.2. Characterization of the Carbonylated Proteome in the Cerebellum: Protein Identification and Functional Enrichment Analysis

Considering that protein carbonylation is a highly selective PTM, the pool of cerebellar proteins vulnerable to carbonylation was investigated. The study of the carbonylated proteome in the cerebellum revealed a total of 59 carbonylated spot targets. Among them, 33 were in the cytosolic protein fraction (Figure S1), and 26 were in the myofibrillar fraction (Figure S2). These spots correspond to 39 different carbonylated proteins identified in all the rats. This defines a protein carbonylation pattern shared by all dietary groups. Protein identifications are shown in Table S4.

**Figure 1:**
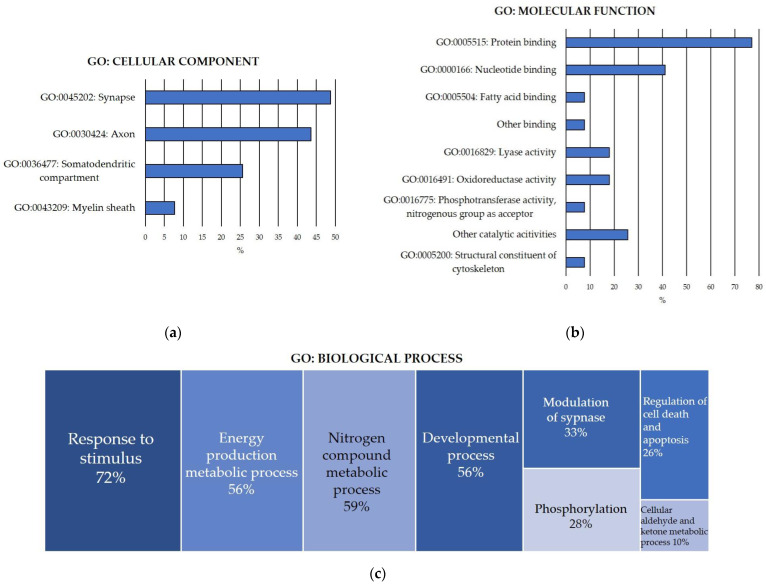
GO Functional enrichment analysis for carbonylated proteins identified in the cerebellum. (**a**) Significantly enriched GO cellular component; (**b**) significantly enriched GO molecular function; (**c**) significantly enriched GO biological process. Analyses were conducted using STRING. FDR: False Discovery Rate. Significant enrichment at FDR < 5%.

To functionally characterize the pool of carbonylated proteins discovered in the rat cerebellum, functional enrichment analyses for the GO terms of cellular component, molecular function and biological process (Figure 1) were conducted.

GO cellular component enrichment analysis of the carbonylated proteins identified in the cerebellum (Figure 1a) revealed that 44% of carbonylated proteins were axonal proteins, particularly from the distal axon, the axon terminus and the terminal bouton. Additionally, 26% of the carbonylated proteins were somatodendritic proteins, mostly located in the neuronal body. Moreover, 8% of the proteins were found in the myelin sheath. Notably, 49% of the carbonylated proteins were specifically components of the synapse, including pre- and postsynaptic cellular components. Interestingly, the results reflected a significant enrichment in proteins from the glutamatergic synapse in the cerebellum.

Furthermore, the analysis demonstrated that 72% of the carbonylated proteins were located in the cytosol, 28% in the cytoskeleton and 31% in the mitochondrion. There was a significant enrichment in proteins found in the cell cortex (15%). Additionally, proteins belonging to two different protein complexes—the mitochondrial pyruvate dehydrogenase and the phosphopyruvate hydratase complexes—were significantly enriched.

Regarding the GO molecular function enrichment analysis of the carbonylated proteins identified in the cerebellum (Figure 1b), the results revealed that 92% of the proteins exhibited binding functions. Among them, 77% were capable of binding to other proteins, showing significant enrichments in identical protein binding and enzyme binging molecular functions. Furthermore, 41% of the carbonylated proteins could bind to nucleotides, with significant enrichment in proteins binding to ATP, ADP and NAD. Additionally, 8% exhibited fatty acid binding capacity. Other significantly enriched binding functions included carboxylic acid binding (13%) and magnesium ion binding (13%). Cerebellar carbonylated proteins were predominantly catalytical enzymes, displaying significant enrichments in diverse lyase activities (18%), oxidoreductases (18%) and phosphotransferases that use nitrogenous group as acceptor (8%).

Another informative analysis shedding light on the role of protein carbonylation in the cerebellum was the GO biological process enrichment analysis. The total pool of carbonylated protein identified in the cerebellum was implicated in over one hundred diverse biological processes, grouped and summarized in Figure 1c. In accordance with this GO term, the cerebellar carbonylated proteome was mainly enriched in (a) the response to stimuli (72%), especially stress and organic molecules like lipids, hormones and cytokines, particularly interleukin-7 (IL-7); (b) energy production, highlighting proteins devoted to carbohydrate metabolism, glycolysis and gluconeogenesis, pyruvate metabolism and phosphocreatinine biosynthesis (56%); (c) nitrogen compound metabolic processes, specifically glutamate metabolism (59%); (d) developmental processes (56%), precisely neuron system development, including neuron differentiation, neuron projection morphogenesis and axonogenesis; (e) modulation of synapse, by regulating transport, organelle localization and synaptic vesicle cycle (33%); (f) regulation of cell death and apoptosis (26%); (g) phosphorylation (28%); and (h) cellular metabolism of aldehydes and ketones (10%).

**Figure 2:**
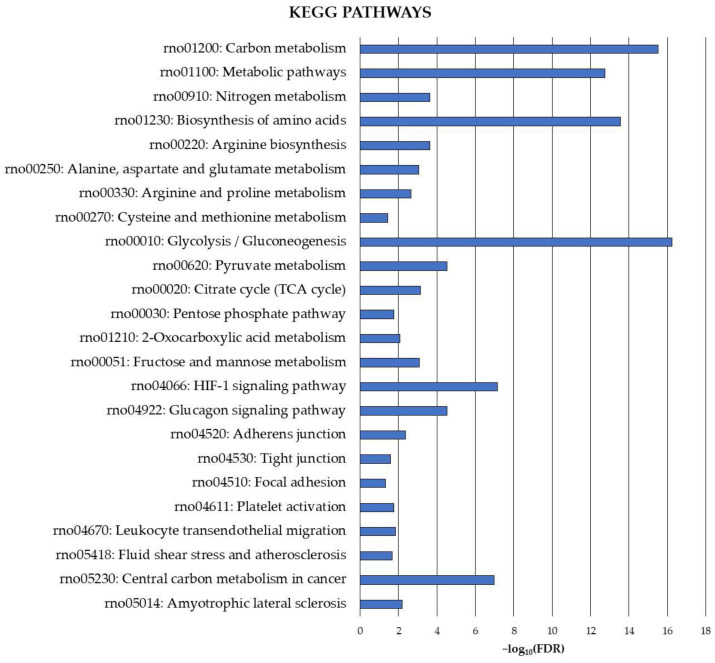
KEGG functional enrichment analysis for carbonylated proteins identified in the cerebellum. Analyses were conducted using STRING. FDR: False Discovery Rate. Significant enrichment at FDR < 5%.

Finally, the exploration of the carbonylated proteome in the rat cerebellum was complemented by the identification of precise pathways potentially under the control of this oxidative protein modification. Therefore, KEGG pathway functional enrichment analysis was also conducted for the cerebellar carbonylated proteins identified in the current study, and the results are shown in Figure 2.

The analysis revealed several cerebellar metabolic pathways potentially modulated by the carbonylation status of the involved proteins. These pathways were mainly related to carbon metabolism and, to a lesser extent, nitrogen metabolism. They played a critical role in amino acid metabolism (biosynthesis of amino acids, arginine biosynthesis, alanine, aspartate and glutamate metabolism, arginine and proline metabolism, cysteine and methionine metabolism) and energy production (glycolysis/gluconeogenesis, pyruvate metabolism, citrate cycle (TCA cycle), pentose phosphate pathway, 2-oxocarboxylic acid metabolism, fructose and mannose metabolism).

Carbonylation also appeared to play a crucial role in regulating signaling pathways in the cerebellum, as evidenced by significant enrichments in proteins belonging to the HIF-1 signaling pathway and the glucagon signaling pathway. Moreover, pathways essential for maintaining the integrity of the BBB at cerebellar level, such as adherens junction, tight junction and focal adhesion, as well as others like platelet activation, leukocyte transendothelial migration and fluid shear stress and atherosclerosis, exhibited significant enrichment in carbonylated proteins. Finally, proteins directly involved in the etiology of neurodegenerative diseases, such as amyotrophic lateral sclerosis and cancer, were identified as targets of carbonylation in the cerebellum.

### 3.3. Effects of Long-Term Feeding of High-Fat and High-Sucrose Diet on Protein Carbonylation Patterns in the Cerebellum

The cerebellar elevation of total protein carbonylation induced by the intake of the HFHS diet (Table 1) raises questions concerning the specific targets of this increased carbonylation. Interestingly, the pro-oxidant effect of the intake of the HFHS diet seems particularly selective regarding cerebellar proteins (Table 2). Thereby, the HFHS diet significantly increased carbonylation of seven proteins out of the pool of carbonylated proteome, constituting 18% of the total. These proteins were fructose-bisphosphate aldolase C (ALDOC), alpha-enolase (ENO1), phosphoglycerate mutase 1 (PGAM1), ATP synthase subunit beta mitochondrial (ATP5F1B), carbonic anhydrase 2 (CA2), creatine kinase B-type (CKB) and creatine kinase U-type mitochondrial (CKMT1). Our previous study in the cerebral cortex of these rats [19] demonstrated that 32% of the carbonylated proteome increased carbonylation due to the HFHS diet. Therefore, a higher proportion of carbonylated proteins are sensitive to the pro-oxidant effect of the HFHS diet in the cerebral cortex than in the cerebellum.

The functional enrichment analyses of the proteins that were significantly more oxidized in the HFHS-fed rats provided valuable insights into the potential effect of the obesogenic diet on the cerebellum.

Firstly, the HFHS diet did not target any specific cellular compartment, but rather molecular functions. Thus, the molecular functions of creatine kinase and lyase activities showed significant enrichment in proteins sensitive to the HFHS diet.

Secondly, the ATP metabolic process, and consequently the ATP homeostasis, seemed to be seriously affected by the HFHS diet in the cerebellum. Accordingly, there were significant enrichments in proteins participating in glycolysis/gluconeogenesis, a critical process for neurons to obtain ATP from glucose—their sole substrate in normal conditions [57]. Furthermore, the HFHS diet significantly increased oxidative damage to both cytosolic and mitochondrial creatine kinase (CK) isoforms, enzymes involved in the rapid ATP production to meet urgent or high neuronal ATP demands [58]. Increased oxidative damage of the cytosolic CK but not the mitochondrial CK was found in the cerebral cortex of these rats fed the HFHS diet [19]. Previous studies have demonstrated that the carbonylation of both cytosolic [59] and mitochondrial [60] CK isoforms decreases their activity and leads to mitochondrial dysfunction. Notably, the cerebellum is a “hot spot” of CK localization in the CNS [61], underscoring the pivotal role of these enzymes in normal cerebellar function. It has been reported that correct ATP–phosphocreatine exchange is critical for maintaining ATP-homeostasis and actin dynamics in growing neuronal dendrites in mouse cerebellar Purkinje cells [62].

Other processes and pathways were significantly enriched in proteins sensitive to the HFHS diet. These included the biosynthesis of amino acids, especially the arginine and proline metabolism, the cellular response to IL-7 and the HIF-1 signaling pathway.

The significant oxidative damage to proteins participating in arginine and proline metabolism could potentially instigate multiple alterations in the HFHS-fed rats. Changes in central metabolites of arginine and proline metabolism could affect both ATP production through TCA and neurotransmission by altering the glutamate metabolism, which is the chief excitatory neurotransmitter [63]. The antioxidant defense could also be affected because glutamate contributes to the synthesis of glutathione, a key antioxidant in the CNS [64]. Additionally, the antioxidant defense could be affected by changes in polyamines, which are end products of arginine and proline metabolism. This is because it has been described that polyamines can protect against ROS, acting as natural free radical scavengers [65]. Lastly, the arginine and proline metabolism seem to have a central role in the evolution of neuropathologies such as amyotrophic lateral sclerosis. This severe condition is characterized by motor neuron degeneration and associated with systemic metabolic impairment [66], including cerebellar pathology [67]. The biosynthesis of amino acids, and particularly glutamate metabolism, was affected by the HFHS diet in the cerebral cortex of these rats as well [19].

Regarding the alteration in proteins related to the cellular response to IL-7, further investigation into the consequences for the cerebellum is needed. IL-7 is a key driver of T-cell homeostasis and function [68], but also could be relevant for the cerebellum. It has been reported that IL-7 exhibits direct neurotrophic properties in rat cerebellum cell culture, suggesting its potential role as a neuronal growth factor with physiological significance during CNS ontogeny [69].

**Figure 3:**
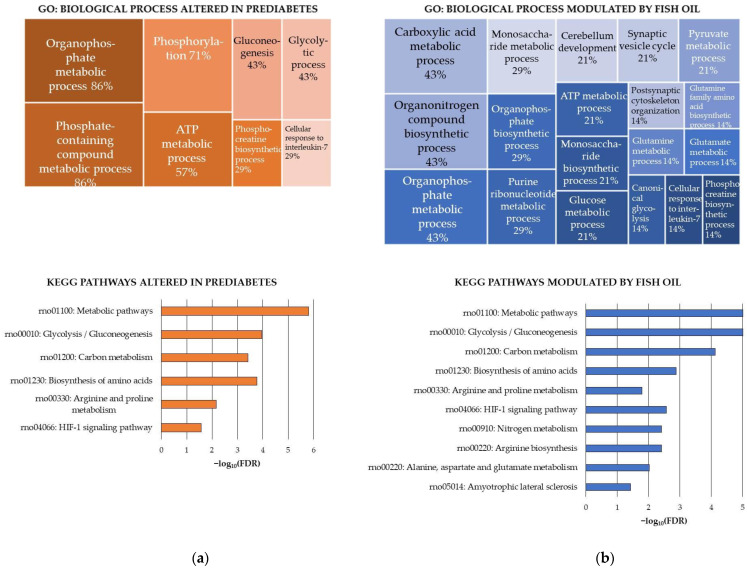
KEGG pathways modulated in a rat model of diet-induced prediabetes and effect of the fish oil supplementation through changes induced on carbonylated proteins in the cerebellum. (**a**) KEGG pathways modulated by the intake of the high-fat and high-sucrose diet (HFHS) and (**b**) KEGG pathways modulated by the fish oil supplementation. Analyses were conducted using STRING. FDR: False Discovery Rate. Significant enrichment at FDR < 5%.

Finally, the HIF-1 signaling pathway seemed to be influenced by the HFHS diet in the cerebellum. Similarly, the HFHS diet increased the carbonylation of proteins involved in this pathway in the cerebral cortex [19]. Previous studies have associated changes in HIF-1 levels in the brain with high-fat diets and brain damage [70]. Details of the GO biological processes and KEGG pathways found enriched in the carbonylated proteins sensitive to the HFHS diet are shown in Figure 3a.

### 3.4. Effects of Fish Oil Supplementation on Protein Carbonylation Patterns in the Cerebellum

As shown in Table 2, 25 spots corresponding to 14 cerebellar carbonylated proteins (36% of the total carbonylated proteome) were sensitive to fish oil supplementation, irrespective of the background diet. These proteins were: ACTB, AKR1A1, ATP5F1B, CKB, CKMT1, ENO1, GAPDH, GLUD1, GLUL, NEFL, PACSIN1, PDHA1, SEPTIN11 and SYN1. Therefore, the redox effect of fish oil was much more diverse in terms of protein targets than the effect of the HFHS diet. A similar outcome was observed in the cerebral cortex of the rats, where almost 44% of the carbonylated proteome responded to fish oil supplementation [19].

The results of the GO functional enrichment analysis indicated that the subset of carbonylated proteins responsive to fish oil supplementation in the cerebellum mainly comprised axonal proteins (50%), primarily from the distal axon, and mitochondrial proteins (43%). These proteins were preferentially located in the synapse (64%), especially the glutamatergic synapse (29%). Several enrichments in postsynapse compartments, including postsynaptic specialization and postsynaptic cytoskeleton, were revealed. Accordingly, there was a significant enrichment in proteins with the molecular function of being structural constituents of postsynapse. Other molecular functions that were significantly enriched were binding to either nucleotide, mainly ATP, identical proteins and enzymes and catalytic activities, especially creatine kinase.

Overall, the effect of the fish oil was sustained in both dietary contexts. Nevertheless, the background diet influenced the magnitude and even direction of the changes caused by the supplement on certain target proteins. Particularly, ten proteins exhibited significantly lesser carbonylation in fish oil-supplemented groups than controls (AKR1A1, ATP5F1B, CKB, CKMT1, GAPDH, GLUD1, GLUL, PDHA1, SEPTIN11 and SYN1). These proteins were generally more sensitive to fish oil in the HFHS dietary background, except for AKR1A1, which was more sensitive in the STD dietary background.

In contrast to these proteins, ENO1 seemed to slightly increase carbonylation due to the fish oil supplementation in both STD and HFHS frameworks as compared to control groups. Despite this, there were no significant differences between groups in the post hoc pairwise comparison analysis. Similarly, ACTB showed a significant increase in carbonylation due to fish oil supplementation, a phenomenon previously documented in rat livers [56]. Nonetheless, actin is a natural free radical scavenger, and moderate oxidative damage to the protein usually has a lower impact on its molecular function [71].

Finally, the background diet had a strong influence on the fish oil’s effect on the carbonylation of NEFL and PACSIN1. In the STD diet, fish oil increased carbonylation of both proteins. In the HFHS diet, fish oil decreased their carbonylation. The observed rise in carbonylation in the STD context might be a response to the slight oxidative stress increase caused by fish oil in the healthy context, as part of its antioxidant mechanism [22,56].

It should be noted that the incorporation of fish oil into the HFHS diet partially counteracted the prooxidant effect measured in the HFHS control rats. This was evident in the two isoforms of creatine kinase and the mitochondrial subunit beta of ATP synthase, which experienced a significant drop in carbonylation level as compared to controls. In the cerebral cortex of these rats, fish oil supplementation was able to almost perfectly counteract the effects of the HFHS diet on the carbonylated proteome. As a result, fish oil significantly prevented oxidative damage to cortical proteins involved in glucose metabolism and neurotransmission [19].

On the other hand, the functional enrichment analysis of the fish oil-sensitive proteins (Figure 3b, upper panel) resulted in a long list of 26 significantly enriched GO biological process terms. Overall, 64% of the proteins participated in the regulation of biological quality. More specifically, the effect of the fish oil was directed toward proteins involved in (a) metabolic processes related to carboxylic acids (43%) (concretely glutamine, glutamate, pyruvate), monosaccharides (29%) (particularly glucose), purine ribonucleotides (29%) (specifically ATP) and phosphocreatine (14%); (b) synapse (29%), particularly postsynaptic cytoskeleton organization and synaptic vesicle cycle; (c) cerebellum development (21%); and (d) cellular response to IL-7 (14%). The functional enrichment analysis conducted using the KEGG pathways database (Figure 3b, bottom panel) confirmed the influence of fish oil on glycolysis/glucogenesis and revealed other specific pathways modulated by ꞷ-3 PUFAs, which were the biosynthesis of amino acids—especially the arginine and proline metabolism, the arginine biosynthesis and the alanine, aspartate and glutamate metabolism—and the HIF-1 signaling pathway.

Several conclusions can be drawn from these data. Firstly, fish oil influenced the same pathways and processes as the HFHS diet but significantly decreased carbonylation of the involved proteins. Remarkably, fish oil could help restore ATP homeostasis in the rat cerebellum by counteracting carbonylation of proteins involved in ATP production, including glycolytic proteins and both isoforms of CK. Additionally, fish oil counteracted oxidative insults caused by the obesogenic diet in proteins related to the cellular response to IL-7, HIF-1 signaling pathway and the arginine and proline metabolism.

Secondly, fish oil supplementation had a stronger influence on the subset of carbonylated proteins participating in the amino acid metabolism as compared to the HFHS diet. Thus, fish oil additionally decreased carbonylation of proteins involved in the arginine biosynthesis and the alanine, aspartate and glutamate metabolism in the cerebellum. This suggested a great influence of fish oil on neurotransmission, especially concerning glutamate and aspartate. Data indicated that fish oils modulate carbonylation levels of proteins from different pathways that cooperate in controlling cell levels of glutamate in the cerebellum. As mentioned earlier, glutamate is the main excitatory neurotransmitter but also the precursor of glutathione, critical for antioxidant defense in the CNS. Intervention with compounds with antioxidant and anti-inflammatory properties, such as gastrodin, promoted glutamate signaling, which was deteriorated in Purkinje cells due to diabetes [27]. Moreover, the decreased carbonylation of proteins that control biological processes devoted to cytoskeletal organization and synaptic vesicle cycle reinforced the capacity of fish oil to modulate neurotransmission.

## 4. Conclusions

The antioxidant properties of fish oil supplementation were investigated in the cerebellum, focusing on its capacity to modulate the carbonylated proteome in rats fed STD or HFHS diets. The most potent antioxidant effect of fish oil was observed when it was incorporated into the obesogenic diet. In this dietary context, fish oil not only decreased overall levels of lipid and protein oxidation in the cerebellum but also mitigated systemic oxidative stress and insulin resistance induced by the obesogenic diet. Moreover, fish oil supplementation selectively diminished the carbonylation of proteins that participate in ATP homeostasis and glutamate metabolism, counteracting the effects of the obesogenic diet in the cerebellum. Additionally, fish oil demonstrated a great capacity to decrease carbonylation of proteins involved in amino acid biosynthesis and neurotransmission. In summary, fish oil supplementation appears to enhance the resilience of the cerebellum to oxidative damage induced by stressors such as obesity-related disorders. These findings provide valuable insights into the effect of HFHS diets on redox homeostasis in the cerebellum and support the development of antioxidant-based nutritional interventions to promote cerebellum health.

## Figures and Tables

**Table 1:** Lipid peroxidation products, oxidized proteins and the main polyunsaturated fatty acids measured in the cerebellum of Sprague-Dawley rats fed either a standard (STD) or a high-fat and high-sucrose diet (HFHS), and a weekly dietary supplementation of either soybean oil (control) or fish oil (ω3) ^1^.

	STD-C	STD-ω3	HFHS-C	HFHS-ω3
Conjugated dienes (mmoles hydroperoxide/kg lipid)	6.30 ^a^ (1.37)	5.64 ^a^ (1.77)	6.79 ^a^ (0.32)	6.54 ^a^ (1.21)
HNE (a.u./μg lipid)	643.56 ^a^ (141.17)	627.00 ^a^ (61.32)	750.11 ^ab^ (69.12)	764.89 ^b^ (85.04)
MDA (a.u./μg lipid)	573.00 ^a^ (88.71)	556.00 ^a^ (79.65)	661.56 ^b^ (34.00)	641.22 ^b^ (15.68)
OxPC (a.u./μg lipid)	140.19 ^a^ (10.80)	171.88 ^ab^ (16.81)	199.48 ^b^ (10.01)	170.23 ^ab^ (6.45)
HNE-protein adducts (a.u./μg protein)	22.97 ^b^ (1.00)	24.08 ^ab^ (3.20)	25.40 ^a^ (1.35)	20.53 ^c^ (0.78)
MDA-protein adducts (a.u./μg protein)	20.45 ^ab^ (1.55)	21.35 ^ab^ (2.14)	22.781 ^a^ (3.01)	18.05 ^b^ (1.35)
OxPC-protein adducts (a.u./μg protein)	76.11 ^a^ (5.82)	72.31 ^a^ (0.57)	78.33 ^a^ (8.21)	77.85 ^a^ (1.26)
Total protein carbonylation (a.u./μg protein) *^#^	68.70.1 ^b^ (7.32)	80.62 ^ab^ (12.63)	100.01 ^a^ (9.16)	84.01 ^ab^ (11.50)
Protein carbonylation of cytosolic proteins (a.u./μg protein) *^#^	67.3 ^b^ (2.1)	81.9 ^ab^ (14.4)	104.7 ^a^ (24.4)	79.40 ^b^ (20.6)
Protein carbonylation of myofibrillar proteins (a.u./μg protein) *	70.1 ^b^ (12.5)	79.3 ^ab^ (23.6)	95.3 ^a^ (8.45)	88.6 ^ab^ (5.60)
% ω6-PUFAs (% total fatty acids) *^$^	11.47 ^a^ (0.43)	10.90 ^a^ (0.38)	10.80 ^a^ (0.67)	9.62 ^b^ (0.46)
% ω3-PUFAs (% total fatty acids)	12.80 ^b^ (1.23)	14.04 ^a^ (0.79)	14.34 ^ab^ (1.47)	14.21 ^ab^ (1.56)
ω3/ω6 *^$^	1.12 ^c^ (0.09)	1.29 ^b^ (0.06)	1.33 ^ab^ (0.09)	1.48 ^a^ (0.10)
Total PUFAs (% total fatty acids)	24.27 ^a^ (1.50)	24.93 ^a^ (1.06)	25.14 ^a^ (2.05)	23.83 ^a^ (1.99)

^1^ Two-way ANOVA analyses were conducted. * *p* < 0.05 significant differences given by the factor “diet” (STD and HFHS); ^$^ *p* < 0.05 significant differences given by the factor “supplement” (CONTROL, ω-3). Superscript ^#^ indicates a significant interaction (*p* < 0.05) between the factors diet and supplement. Means with different superscript ^a–c^ indicate significant differences (*p* < 0.05) (analyzed by post hoc Tukey HSD).

**Table 2:** Proteins whose carbonylation level was modulated by the high-fat and high-sucrose diet, the fish oil supplementation or both in the cerebellum of Sprague–Dawley rats ^1^.

				Specific Protein Carbonylation (a.u./μg Protein)			
Spot Nº	Figure	Protein Abbreviation	Protein Description	STD-C	STD-ω3	HFHS-C	HFHS-ω3
2	S2	ACTB	Actin cytoplasmic 1 ^$^	0.14 ^ab^ (0.06)	0.22 ^ab^ (0.06)	0.14 ^b^ (0.08)	0.28 ^a^ (0.05)
23	S1	AKR1A1	Aldo-keto reductase family 1 member A1 ^$#^	0.30 ^a^ (0.09)	0.09 ^b^ (0.03)	0.16 ^ab^ (0.02)	0.13 ^b^ (0.07)
33	S1	ALDOC	Fructose-bisphosphate aldolase C *	0.16 ^a^ (0.06)	0.15 ^a^ (0.04)	0.26 ^a^ (0.08)	0.21 ^a^ (0.01)
4	S2	ATP5F1B	ATP synthase subunit beta mitochondrial *	0.05 ^a^ (0.02)	0.07 ^a^ (0.01)	0.11 ^a^ (0.04)	0.17 ^a^ (0.09)
15	S2	ATP5F1B	ATP synthase subunit beta mitochondrial ^$^	0.17 ^ab^ (0.04)	0.14 ^ab^ (0.05)	0.24 ^a^ (0.06)	0.12 ^b^ (0.02)
33	S1	CA2	Carbonic anhydrase 2 *	0.16 ^a^ (0.06)	0.15 ^a^ (0.04)	0.26 ^a^ (0.08)	0.21 ^a^ (0.01)
3	S1	CKB	Creatine kinase B-type *	0.05 ^a^ (0.02)	0.07 ^a^ (0.01)	0.12 ^a^ (0.08)	0.11 ^a^ (0.04)
7	S2	CKB	Creatine kinase B-type ^$^	0.30 ^ab^ (0.13)	0.22 ^ab^ (0.04)	0.43 ^a^ (0.09)	0.21 ^b^ (0.04)
30	S2	CKMT1	Creatine kinase U-type mitochondrial *^$#^	0.08 ^b^ (0.02)	0.06 ^b^ (0.01)	0.14 ^a^ (0.02)	0.05 ^b^ (0.02)
8	S1	ENO1	Alpha-enolase *^$^	0.02 ^b^ (0.00)	0.04 ^ab^ (0.02)	0.04 ^ab^ (0.01)	0.05 ^a^ (0.01)
24	S1	GAPDH	Glyceraldehyde-3-phosphate dehydrogenase ^$^	0.31 ^a^ (0.01)	0.15 ^b^ (0.07)	0.30 ^a^ (0.05)	0.13 ^b^ (0.04)
9	S2	GAPDH	Glyceraldehyde-3-phosphate dehydrogenase ^$^	0.21 ^a^ (0.06)	0.12 ^a^ (0.07)	0.25 ^a^ (0.05)	0.15 ^a^ (0.03)
10	S2	GAPDH	Glyceraldehyde-3-phosphate dehydrogenase ^$^	0.19 ^a^ (0.03)	0.05 ^b^ (0.01)	0.13 ^a^ (0.01)	0.11 ^ab^ (0.05)
6	S2	GLUD1	Glutamate dehydrogenase 1 mitochondrial ^$^	0.45 ^a^ (0.13)	0.29 ^a^ (0.08)	0.45 ^a^ (0.09)	0.29 ^a^ (0.07)
17	S2	GLUD1	Glutamate dehydrogenase 1 mitochondrial ^$^	0.24 ^a^ (0.12)	0.13 ^a^ (0.02)	0.19 ^a^ (0.03)	0.13 ^a^ (0.03)
7	S2	GLUL	Glutamine synthetase ^$^	0.30 ^ab^ (0.13)	0.22 ^ab^ (0.04)	0.43 ^a^ (0.09)	0.21 ^b^ (0.04)
23	S2	GLUL	Glutamine synthetase ^$^	0.16 ^a^ (0.04)	0.14 ^a^ (0.04)	0.21 ^a^ (0.07)	0.11 ^a^ (0.03)
5	S2	NEFL	Neurofilament light polypeptide ^#^	0.20 ^b^ (0.07)	0.49 ^a^ (0.13)	0.31 ^ab^ (0.12)	0.25 ^ab^ (0.04)
5	S2	PACSIN1	Protein kinase C and casein kinase substrate in neurons protein 1 ^#^	0.20 ^b^ (0.07)	0.49 ^a^ (0.13)	0.31 ^ab^ (0.12)	0.25 ^ab^ (0.04)
7	S2	PDHA1	Pyruvate dehydrogenase E1 component subunit alpha somatic form mitochondrial ^$^	0.30 ^ab^ (0.13)	0.22 ^ab^ (0.04)	0.43 ^a^ (0.09)	0.21 ^b^ (0.07)
23	S2	PDHA1	Pyruvate dehydrogenase E1 component subunit alpha somatic form mitochondrial ^$^	0.16 ^a^ (0.04)	0.14 ^a^ (0.04)	0.21 ^a^ (0.07)	0.11 ^a^ (0.03)
33	S1	PGAM1	Phosphoglycerate mutase 1 *	0.16 ^a^ (0.06)	0.15 ^a^ (0.04)	0.26 ^a^ (0.08)	0.21 ^a^ (0.01)
15	S2	SEPTIN11	Septin-11 ^$^	0.17 ^ab^ (0.04)	0.14 ^ab^ (0.05)	0.24 ^a^ (0.06)	0.12 ^b^ (0.02)
23	S1	SYN1	Synapsin-1 ^$^	0.30 ^a^ (0.09)	0.09 ^b^ (0.03)	0.16 ^ab^ (0.02)	0.13 ^b^ (0.07)
9	S2	SYN1	Synapsin-1 ^$^	0.21 ^a^ (0.06)	0.12 ^a^ (0.07)	0.25 ^a^ (0.05)	0.15 ^a^ (0.03)

^1^ Two-way ANOVA analyses were conducted. * *p* < 0.05 significant differences given by the factor “diet” (STD and HFHS); ^$^ *p* < 0.05 significant differences given by the factor “supplement” (CONTROL, ω-3). Superscript ^#^ indicates a significant interaction (*p* < 0.05) between the factors diet and supplement. Means with different superscript ^a,b^ indicate significant differences (*p* < 0.05) (analyzed by post hoc Tukey HSD). Spot Nº refers to the numbered spots in 2-DE gel images shown in Figures S1 and S2. a.u.: arbitrary units.

## Data Availability

The data presented in this study are available in the article and Supplementary Materials.

## Supplementary Materials

The following supporting information can be downloaded at: https://www.mdpi.com/article/10.3390/antiox13010103/s1, Figure S1: Representative 2-DE gel images showing carbonylated and total proteins identified in the cytosolic fraction of rat cerebellum. Upper panels are the FTSC-labeled 2-DE gel images and bottom panels are the corresponding Coomassie-stained 2-DE gel images from (a) STD-C, (b) STD-ω3, (c) HFHS-C and (d) HFHS-ω3 experimental groups. Numbered protein spots (1–33) indicate carbonylated proteins confidently identified and listed in Table S4. Images are representatives of three independent labeling experiments performed in triplicates. STD: standard diet. HFHS: high-fat and high-sucrose diet; C: control oil (soybean oil), ω3: fish oil.; Figure S2: Representative 2-DE gel images showing carbonylated and total proteins identified in the myofibrillar fraction of rat cerebellum. Upper panels are the FTSC-labeled 2-DE gel images and bottom panels are the corresponding Coomassie-stained 2-DE gel images from (a) STD-C, (b) STD-ω3, (c) HFHS-C and (d) HFHS-ω3 experimental groups. Numbered protein spots (34–59) indicate carbonylated proteins confidently identified and listed in Table S4. Images are representatives of three independent labeling experiments performed in triplicates. STD: standard diet. HFHS: high-fat and high-sucrose diet; C: control oil (soybean oil), ω3: fish oil.; Table S1: Diet composition.; Table S2: Fatty acid composition of each diet.; Table S3. Biometrical and biochemical data from Sprague-Dawley rats fed either a standard diet (STD) or a high-fat and high-sucrose diet (HFHS). Control groups (STD-C and HFHS-C) were supplemented with soybean oil. ω3 groups (STD-ω3 and HFHS-ω3) were supplemented with fish oil.; Table S4. Carbonylated protein identified in rat cerebellum. Nº Spot column numbers are referred to the numbered spots in Figures S1 and S2 (pointed out in column “Figure”). Two-way ANOVA analyses were conducted. * *p* < 0.05 significant differences given by the factor “diet” (STD and HFHS); ^$^ *p* < 0.05 significant differences given by the factor “supplement” (CONTROL, ω3). Superscript ^#^ indicates significant interaction (*p* < 0.05) between the factors diet and supplement. Means with different superscript indicate significant differences (*p* < 0.05) (analyzed by post hoc Tukey HSD).
