# Supplementary material for: Dietary Marine Oils Selectively Decrease Obesogenic Diet-Derived Carbonylation in Proteins Involved in ATP Homeostasis and Glutamate Metabolism in the Rat Cerebellum
Source: Antioxidants (Basel). 2024 Jan 15;13(1):103. doi: 10.3390/antiox13010103 (PMC10812471; doi:10.3390/antiox13010103)
Supplement: Supplementary file 1 [file antioxidants-13-00103-s001.zip › Supplementary Figures S1 and S2.pdf]

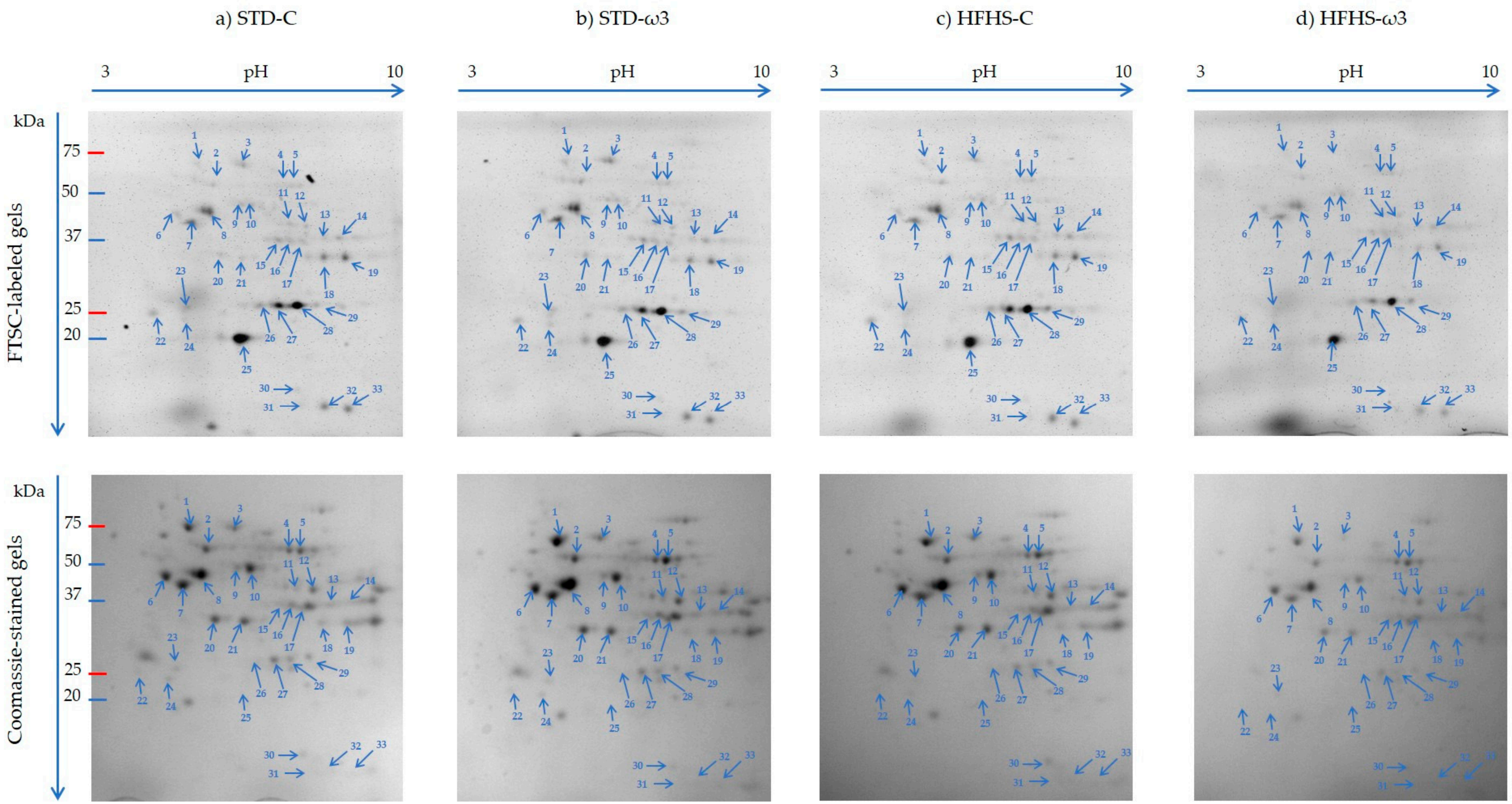

Supplementary Figure S1: Representative 2-DE gel images showing carbonylated and total proteins identified in the cytosolic fraction of rat cerebellum. Upper panels are the FTSC-labeled 2-DE gel images and bottom panels are the corresponding Coomassie-stained 2-DE gel images from a) STD-C, b) STD-ω3, c) HFHS-C and d) HFHS-ω3 experimental groups. Numbered protein spots (1-33) indicate carbonylated proteins confidently identified and listed in Supplementary Table S4. Images are representatives of three independent labeling experiments performed in triplicates. STD: standard diet. HFHS: high-fat and high-sucrose diet; C: control oil (soybean oil), ω3: fish oil.

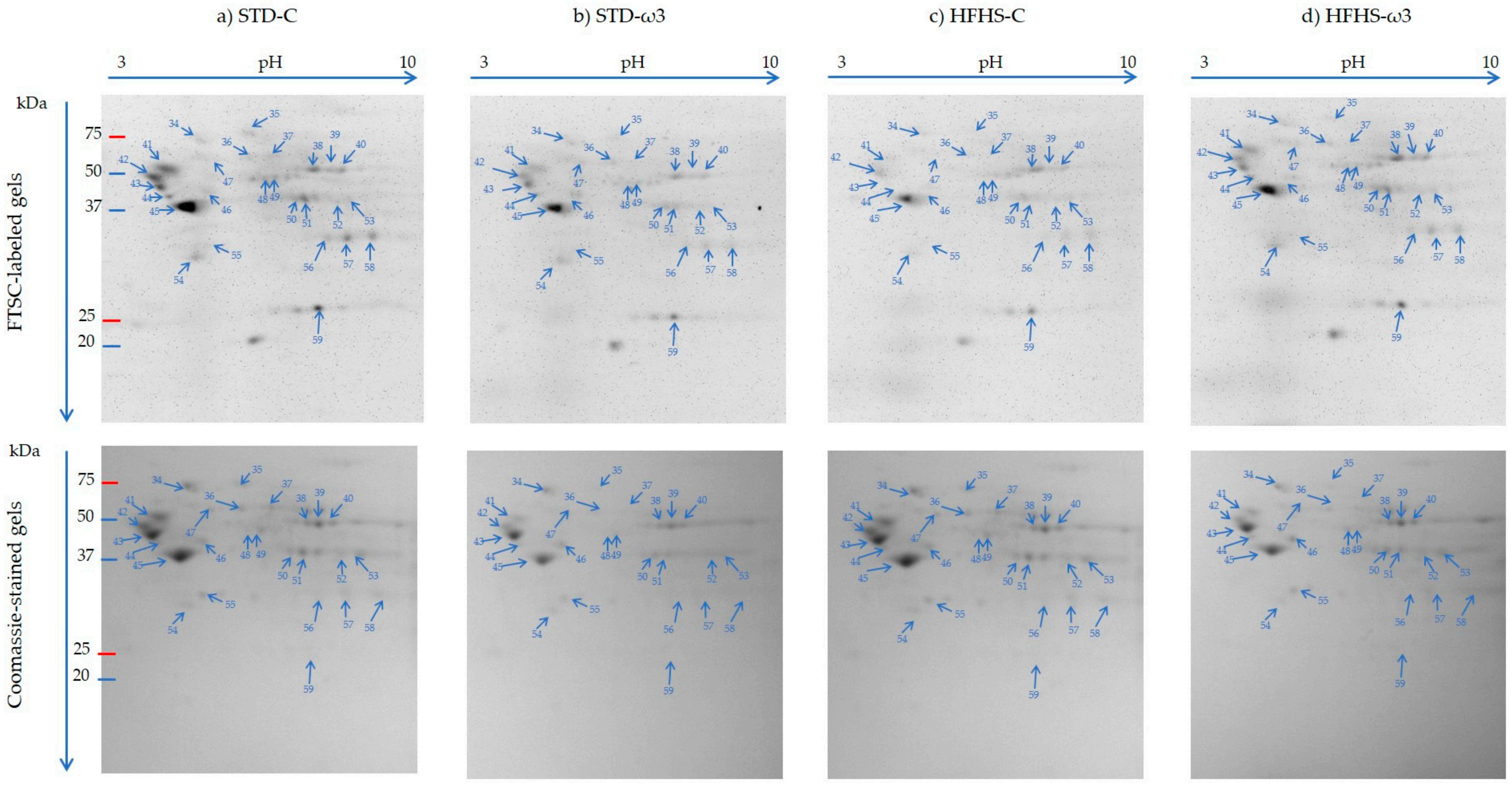

Supplementary Figure S2: Representative 2-DE gel images showing carbonylated and total proteins identified in the myofibrillar fraction of rat cerebellum. Upper panels are the FTSC-labeled 2-DE gel images and bottom panels are the corresponding Coomassie-stained 2-DE gel images from a) STD-C, b) STD-ω3, c) HFHS-C and d) HFHS-ω3 experimental groups. Numbered protein spots (34-59) indicate carbonylated proteins confidently identified and listed in Supplementary Table S4. Images are representatives of three independent labeling experiments performed in triplicates. STD: standard diet. HFHS: high-fat and high-sucrose diet; C: control oil (soybean oil), ω3: fish oil.
