# Supplementary material for: Dietary Marine Oils Selectively Decrease Obesogenic Diet-Derived Carbonylation in Proteins Involved in ATP Homeostasis and Glutamate Metabolism in the Rat Cerebellum
Source: Antioxidants (Basel). 2024 Jan 15;13(1):103. doi: 10.3390/antiox13010103 (PMC10812471; doi:10.3390/antiox13010103)
Supplement: Supplementary file 1 [file antioxidants-13-00103-s001.zip › Supplementary Tables S1-S3.pdf]

Table S1: Diet composition.

	STD-C/-ω3[a*]	HFHS-C/-ω3[b*]
**Protein (%)**	14.3	17.3
**Fat (%)**	4.0	23.2
**Anhydrous Milkfat (%)**		21
**Carbohydrate (available) (%)**	48.0	47.6
**Sucrose (%)**	-	34
**Fiber (%)**	22.1	5
**Minerals (%)**	4.7[c]	4.3[d]
**Vitamins (%)**	1.2[e]	1.9[f]
**Total energy density (kcal/g)**	2.9	4.7
**Calories from Protein (%)**	20	14.7
**Calories from Fat (%)**	13	44.6
**Calories from Carbohydrates (%)**	67	40.7

[a]Teklad Global 14% Protein Rodent Maintenance Diet (Envigo, IN, USA).

[b]TD.08811 45% Kcal Fat Diet (Envigo, IN, USA).

[c]Calcium (0.7%), phosphorus (0.6%), non-phytate phosphorus (0.3%), sodium (0.1%), potassium (0.6%), chloride (0.3%), magnesium (0.2%), zinc (70 mg/kg), manganese (100 mg/kg), copper (15 mg/kg), iodine (6 mg/kg), iron (175 mg/kg), selenium (0.23 mg/kg).

[d]43 g/kg chow of Mineral Mix, AIN-93G-MX (94046): Calcium Carbonate (35.7%), monopotassium phosphate (19.6%), potassium citrate monohydrate (7.1%), sodium chloride (7.4%), potassium sulfate (4.7%), magnesium oxide (2.4%), ferric citrate (0.6%), zinc carbonate (0.2%), manganese carbonate (0.06%), copper carbonate (0.03%), potassium iodate (0.001%), sodium selenate anhydrous (0.001%), ammonium molybdate.4H2O (0.0008%), sodium metasilicate.9H2O (0.15%), chromium potassium sulfate.12H2O (0.3%), lithium chloride (0.002%), boric acid (0.008%), sodium fluoride (0.006%), nickel carbonate (0.003%), ammonium vanadate (0.0007%), powdered sugar (22.1%).

[e]Vitamin A (17 IU/g), vitamin D3 (1.2 IU/g), vitamin E (150 IU/kg), vitamin K3 (menadione; 58 mg/kg), vitamin B1 (thiamin; 64 mg/kg), vitamin B2 (riboflavin; 16 mg/kg), niacin (nicotinic acid; 84 mg/kg), vitamin B6 (pyridoxine; 17 mg/kg), pantothenic acid (76 mg/kg), vitamin B12 (cyanocobalamin, 0.09 mg/kg), biotin (0.57 mg/kg), folate (5 mg/kg), choline (1030 mg/kg).

[f]3 g/kg choline bitartrate + 19 g/kg chow of Vitamin Mix, AIN-93-VX (94047): nicotinic acid (0.3%), D-calcium pantothenate (0.16%), pyridoxine HCl (0.07%), thiamine HCl (0.06%), riboflavin (0.06%), folic acid (0.02%), D-biotin (0.002%), vitamin B12 (0.01% triturated in mannitol) (0.25%), a-tocopherol powder (250 U/g; 3%), vitamin A palmitate (250,000 U/g; 0.16%), vitamin D3 (400,000 U/g; 0.025%), phylloquinone (0.0075%), powdered sucrose (95.97%).

*STD-C and HFHS-C groups received a weekly oral dose of 0.8 ml/kg body weight of soybean oil; STD-ω3 and HFHS-ω3 groups received a weekly oral dose of 0.8 ml/kg body weight of fish oil.

Table S2: Fatty acid composition of each diet.

Fatty acid*	STD-C[a]	STD-ω3[b]	HFHS-C[a]	HFHS-ω3[b]
**14:0**	0.0	0.1	11.5	11.6
**16:0**	14.8	14.6	32.2	32.0
**16:1ω7**	0.0	0.1	1.7	1.7
**18:00**	2.9	2.9	13.7	13.7
**18:1ω9**	20.5	20.3	26.4	26.2
**18:1ω7**	0.0	0.0	0.7	0.7
**18:2ω6**	58.6	57.8	9.7	8.7
**20:0**	n.d.	0.0	n.d.	0.0
**18:3ω3**	2.9	2.9	1.1	1.1
**20:1ω9**	0.0	0.0	0.0	0.0
**18:4ω3**	0.0	0.0	0.0	0.0
**20:2ω6**	0.0	0.0	0.0	0.0
**20:3ω6**	n.d.	0.0	n.d.	0.0
**20:4ω6**	0.0	0.0	0.0	0.0
**22:1ω11**	0.0	0.0	0.0	0.0
**22:1ω9**	0.0	0.0	0.0	0.0
**20:4ω3**	0.0	0.0	0.0	0.0
**20:5ω3**	0.0	0.4	0.0	0.5
**24:1ω9**	0.0	0.0	0.0	0.0
**22:5ω3**	0.0	0.1	0.0	0.1
**22:6ω3**	0.0	0.4	0.0	0.6
**Total SFAs**	**17.5**	**18.3**	**59.4**	**60.5**
**Total MUFAs**	**21.1**	**20.3**	**29.3**	**28.4**
**Total PUFAs**	**61.1**	**61.0**	**10.8**	**10.7**
**Total ω3**	**3.3**	**3.2**	**1.6**	**1.4**
**Total ω6**	**58.8**	**58.9**	**10.4**	**10.6**

*Results are expressed as a percentage of total fatty acids (mg/100mg of Total FA).
[a]STD-C and HFHS-C groups received a weekly oral dose of 0.8 ml/kg body weight of soybean oil.
[b]STD-ω3 and HFHS- ω3 groups received a weekly oral dose of 0.8 ml/kg body weight of fish oil.

Table S3. Biometrical and biochemical data from Sprague-Dawley rats fed either a standard diet (STD) or a high-fat and high-sucrose diet (HFHS). Control groups (STD-C and HFHS-C) were supplemented with soybean oil. ω3 groups (STD-ω3 and HFHS-ω3) were supplemented with fish oil.[1,2]

	STD-C	STD-ω3	HFHS-C	HFHS-ω3
	**Mean (SD)**	**Mean (SD)**	**Mean (SD)**	**Mean (SD)**
**% of initial body Weight**^{z*}	114.07^{a} (2.46)	115.47^{ab} (3.27)	118.43^{b} (3.69)	115.90^{ab} (3.69)
**Adiposity Index (%)**^{y*}	1.67^{a} (0.44)	1.65^{a} (0.38)	2.37^{b} (0.75)	2.32^{b} (0.69)
**% Fat in liver**$^{y*\$}$	7.01^{a} (0.51)	6.36^{b} (0.28)	7.34^{a} (0.45)	6.55^{b} (0.38)
**% Fat in kidney**$^{y*\$}$	4.31^{a} (0.23)	4.11^{ab} (0.24)	5.65^{a} (0.56)	4.39^{b} (0.39)
**% Fat in adipose tissue**$^{y*\$}$	96.32^{a} (2.05)	92.73^{b} (3.16)	99.51^{a} (2.03)	97.01^{b} (1.20)
**Plasma Insulin ng/mL**^{x*}	0.56^{a} (0.32)	0.65^{a} (0.19)	1.81^{b} (0.82)	1.46^{b} (0.72)
**Plasma Glucose mg/mL** ^{x*}	63.00^{a} (4.84)	63.44^{a} (4.1)	70.78^{a} (4.99)	71.33^{a} (5.32)
**GPx activity (U/g Hb)**	85.41^{a} (14.75)	80.87^{ab} (19.18)	83.45^{a} (13.06)	71.34^{b} (6.82)
**GSSG/GSH in erythrocyte**$^{x*\$\#}$	0.60^{a} (0.25)	1.07^{b} (0.34)	1.48^{b} (1.09)	1.56^{b} (0.87)
**Lipid peroxidation (mmol hydroperoxides/mL plasma)**$^{x\#}$	0.13^{a} (0.03)	0.13^{a} (0.02)	0.21^{a} (0.06)	0.14^{a} (0.02)
**Plasma ORAC (μmol Trolox equivalents/mL)**$^{\$}$	18.33^{a} (3.76)	19.12^{a} (2.64)	17.41^{a} (4.71)	21.18^{a} (6.12)
**Plasma albumin carbonylation index**$^{x\$\#}$	0.41^{ab} (0.04)	0.33^{ab} (0.05)	0.43^{a} (0.11)	0.32^{b} (0.04)
**Liver carbonylation index**$^{x*\$\#}$	0.27^{a} (0.02)	0.32^{b} (0.03)	0.45^{c} (0.03)	0.28^{a} (0.03)
**Kidney carbonylation index**$^{x*\$\#}$	0.24^{a} (0.01)	0.30^{b} (0.02)	0.43^{c} (0.04)	0.30^{a} (0.03)

[1]Two-way ANOVA analyses were conducted. * $p<0.05$ significant differences given by the factor "diet" (STD and HFHS); $ $p<0.05$ significant differences given by the factor "supplement" (CONTROL, ω-3). Superscript # indicates significant interaction ($p<0.05$) between the factors diet and supplement. Means with different superscript indicate significant differences ($p<0.05$) (analyzed by post-hoc Tukey HSD).

[z]A percentage of the initial body weight during the course of the experiment. Body weight percentage was calculated as (final body weight/initial body weight) × 100.

[y]Adiposity index: (total abdominal fat×100)/body weight.

ORAC: oxygen radical absorbance capacity. GPx: Glutathione peroxidase. GSSG/GSH: oxidized/reduced glutathione ratio.

[2] Parameters in the table are already published in:

Dasilva, G; Lois, S.; Méndez, L.; Miralles-Pérez, B.; Romeu, M.; Ramos-Romero, S.; Torres, J.L.; Medina, I. Fish Oil Improves Pathway-Oriented Profiling of Lipid Mediators for Maintaining Metabolic Homeostasis in Adipose Tissue of Prediabetic Rats, Front. Immunol. 12 (2021) 8848161. https://doi.org/10.3389/fimmu.2021.608875.

Méndez, L.; Muñoz, S.; Barros, L.; Miralles-Pérez, B.; Romeu, M.; Ramos-Romero, S.; Torres, J.L.; Medina, I. Combined Intake of Fish Oil and D-Fagomine Prevents High-Fat High-Sucrose Diet-Induced Prediabetes by Modulating Lipotoxicity and Protein Carbonylation in the Kidney, Antioxidants. 12 (2023) 751. https://doi.org/10.3390/antiox12030751.

Hereu, M.; Ramos-Romero, S.; Busquets, C.; Atienza, L.; Amézqueta, S.; Miralles-Pérez, B.; Nogués, M.R.; Méndez, L.; Medina, I.; Torres, J.L. Effects of combined d-fagomine and omega-3 PUFAs on gut microbiota subpopulations and diabetes risk factors in rats fed a high-fat diet. *Sci. Rep.* **2019**, *9*, 16628. https://doi.org/10.1038/s41598-019-52678-5

Hereu, M.; Ramos-Romero, S.; García-González, N.; Amézqueta, S.; Torres, J.L. Eubiotic effect of buckwheat d-fagomine in healthy rats. *J. Funct. Foods* **2018**, *50*, 120–126. https://doi.org/10.1016/j.jff.2018.09.018

Hereu, M.; Ramos-Romero, S.; Marín-Valls, R.; Amézqueta, S.; Miralles-Pérez, B.; Romeu, M.; Méndez, L.; Medina, I.; Torres, J.L. Combined Buckwheat d-Fagomine and Fish Omega-3 PUFAs Stabilize the Populations of Gut Prevotella and Bacteroides While Reducing Weight Gain in Rats. *Nutrients* **2019**, *11*, 2606. https://doi.org/10.3390/nu11112606

Miralles-Pérez, B.; Nogués, M.R.; Sánchez-Martos, V.; Taltavull, N.; Méndez, L.; Medina, I.; Ramos-Romero, S.; Torres, J.L.; Romeu, M. The Effects of the Combination of Buckwheat D-Fagomine and Fish Omega-3 Fatty Acids on Oxidative Stress and Related Risk Factors in Pre-Obese Rats. *Foods* **2021**, *10*, 332. https://doi.org/10.3390/foods10020332

Moreno, F.; Méndez, L.; Raner, A.; Miralles-Pérez, B.; Romeu, M.; Ramos-Romero, S.; Torres, J.L.; Medina, I. Fish Oil Supplementation Counteracts the Effect of High-Fat and High-Sucrose Diets on the Carbonylated Proteome in the Rat Cerebral Cortex. Biomed. Pharmacother. 2023, 168, 115708. https://doi.org/10.1016/j.biopha.2023.115708
